# Supplementary figures and images for: Tracing neuronal circuits in transgenic animals by transneuronal control of transcription (TRACT)
Source: eLife. 2017 Dec 12;6:e32027. doi: 10.7554/eLife.32027 (PMC5777821; doi:10.7554/eLife.32027)

ID3 dNRR dNotch1 TMD dnlng2 esn V5


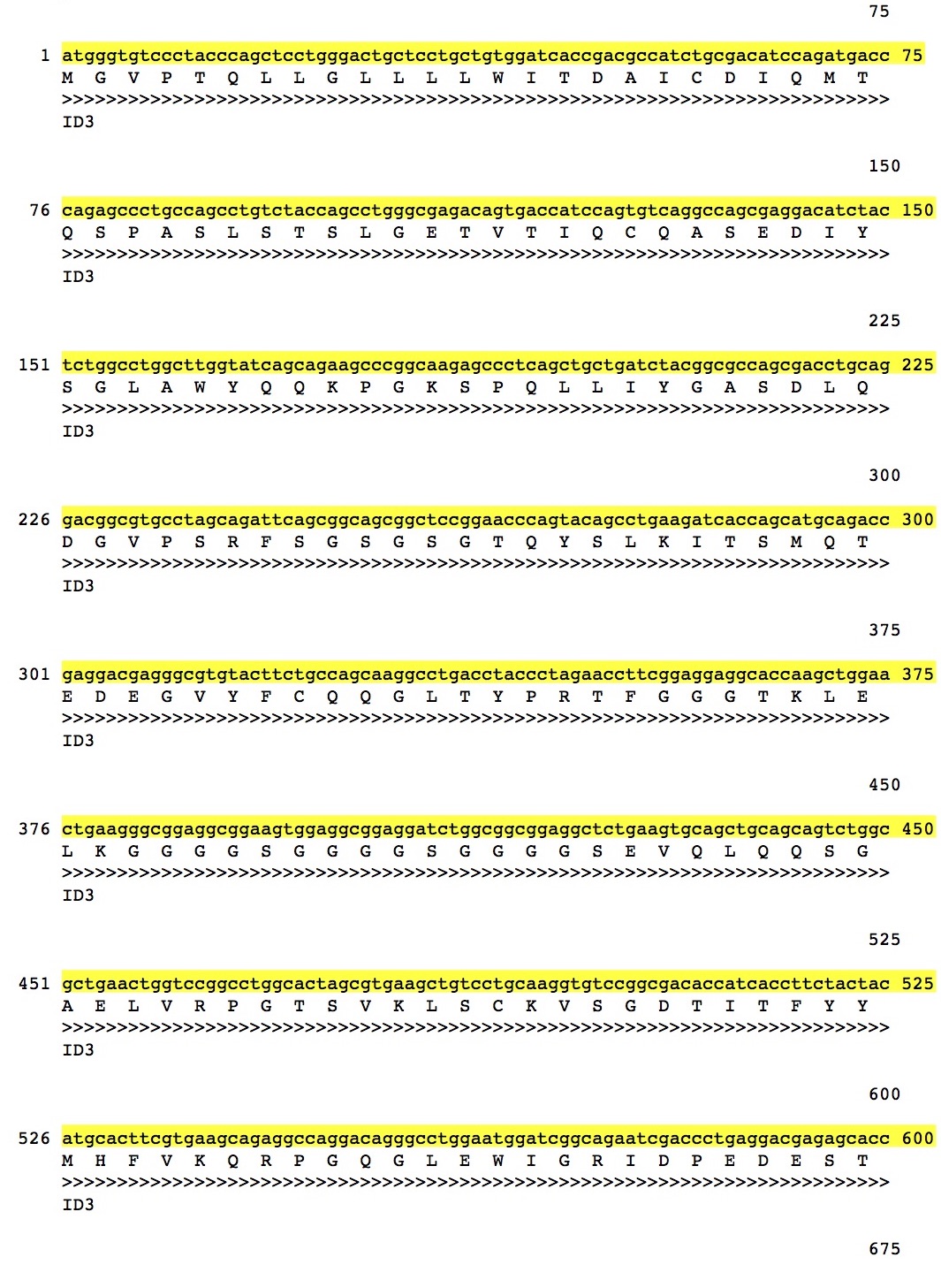


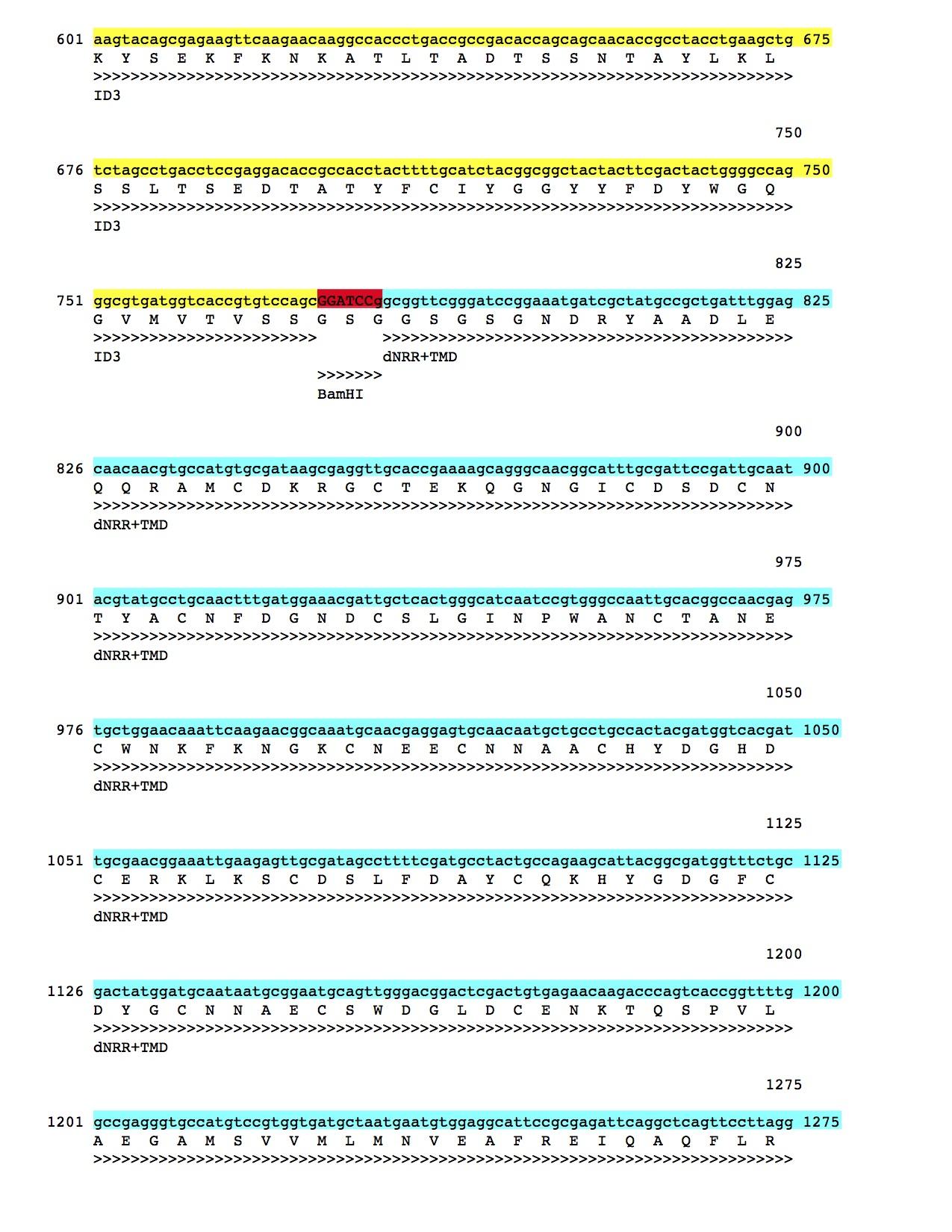

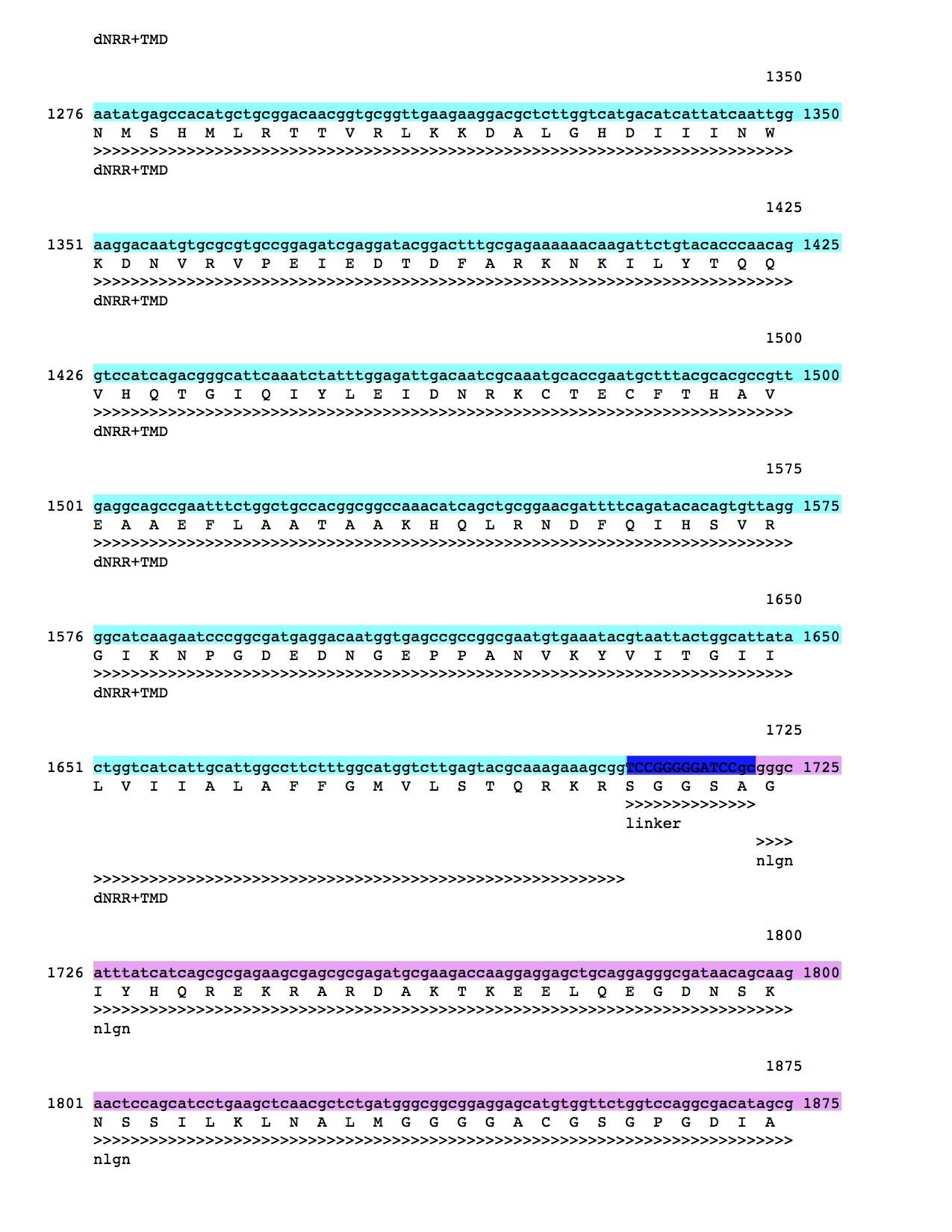


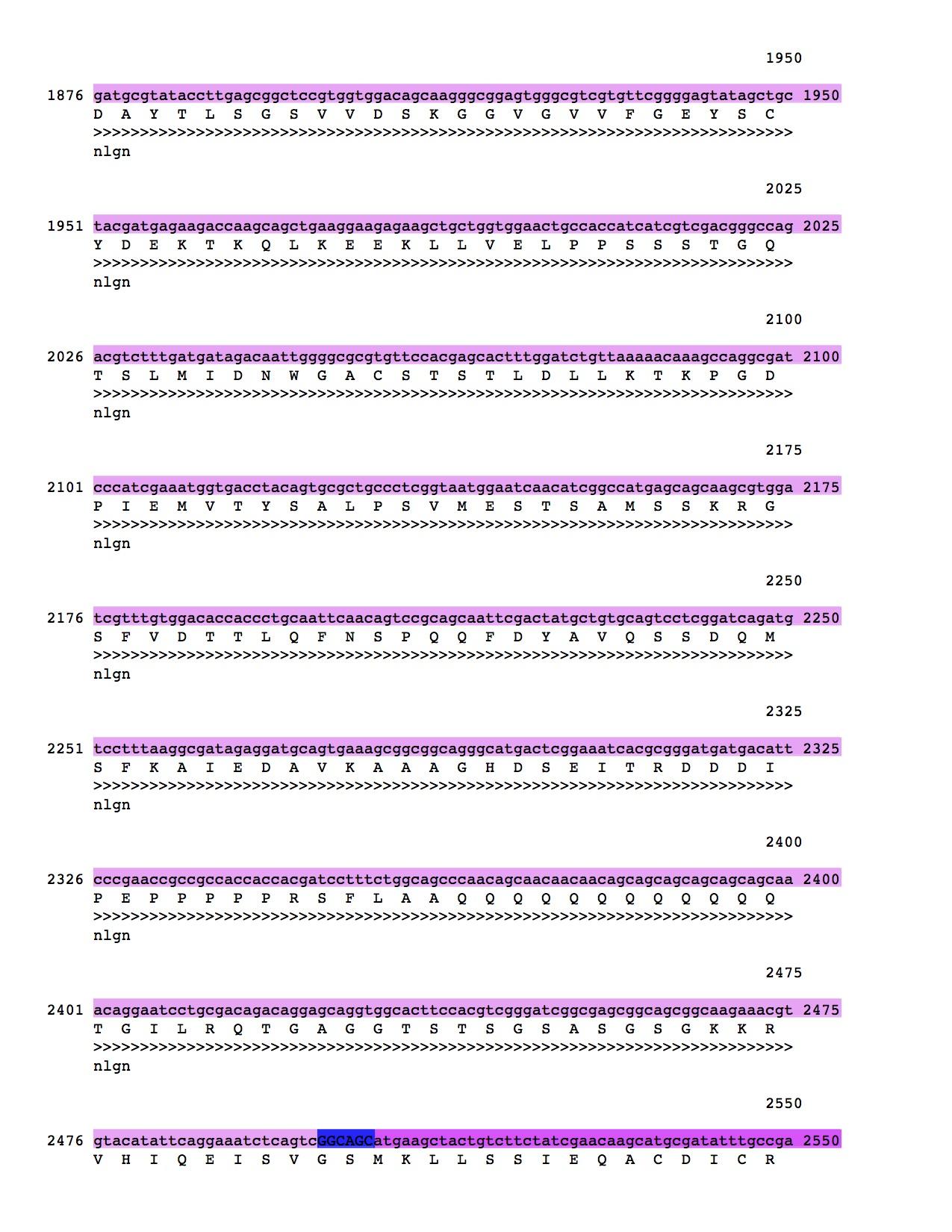


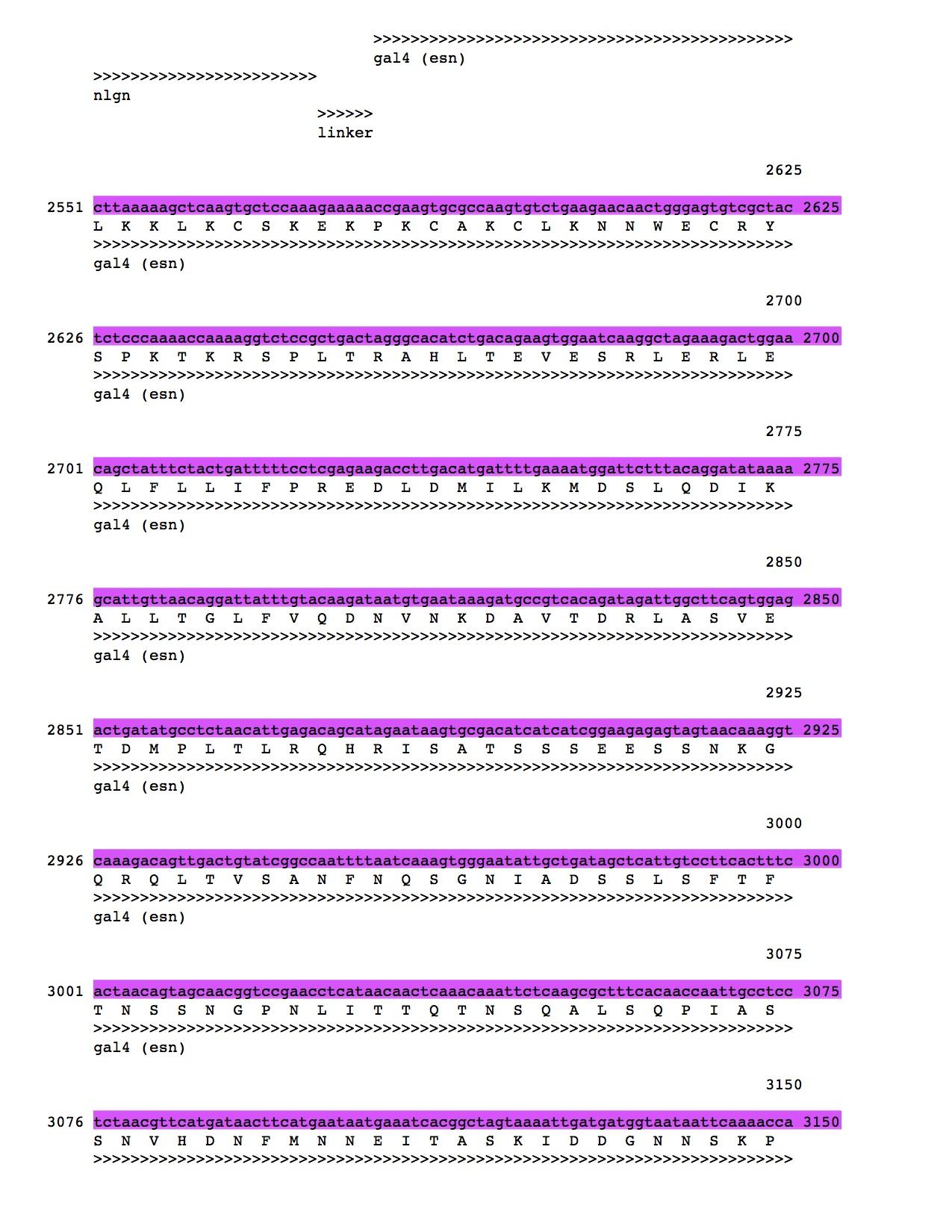


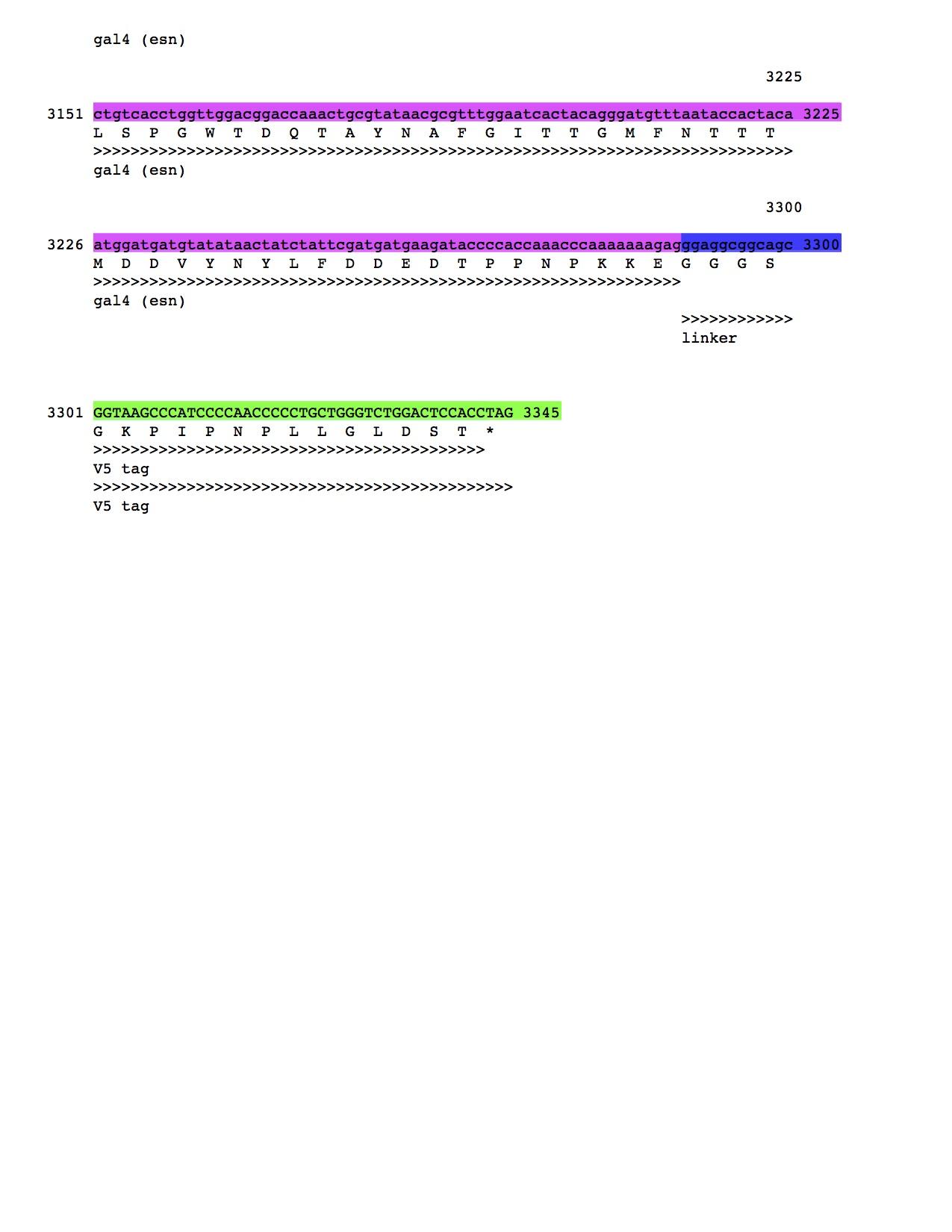


nSyb::CD19 OLLAS


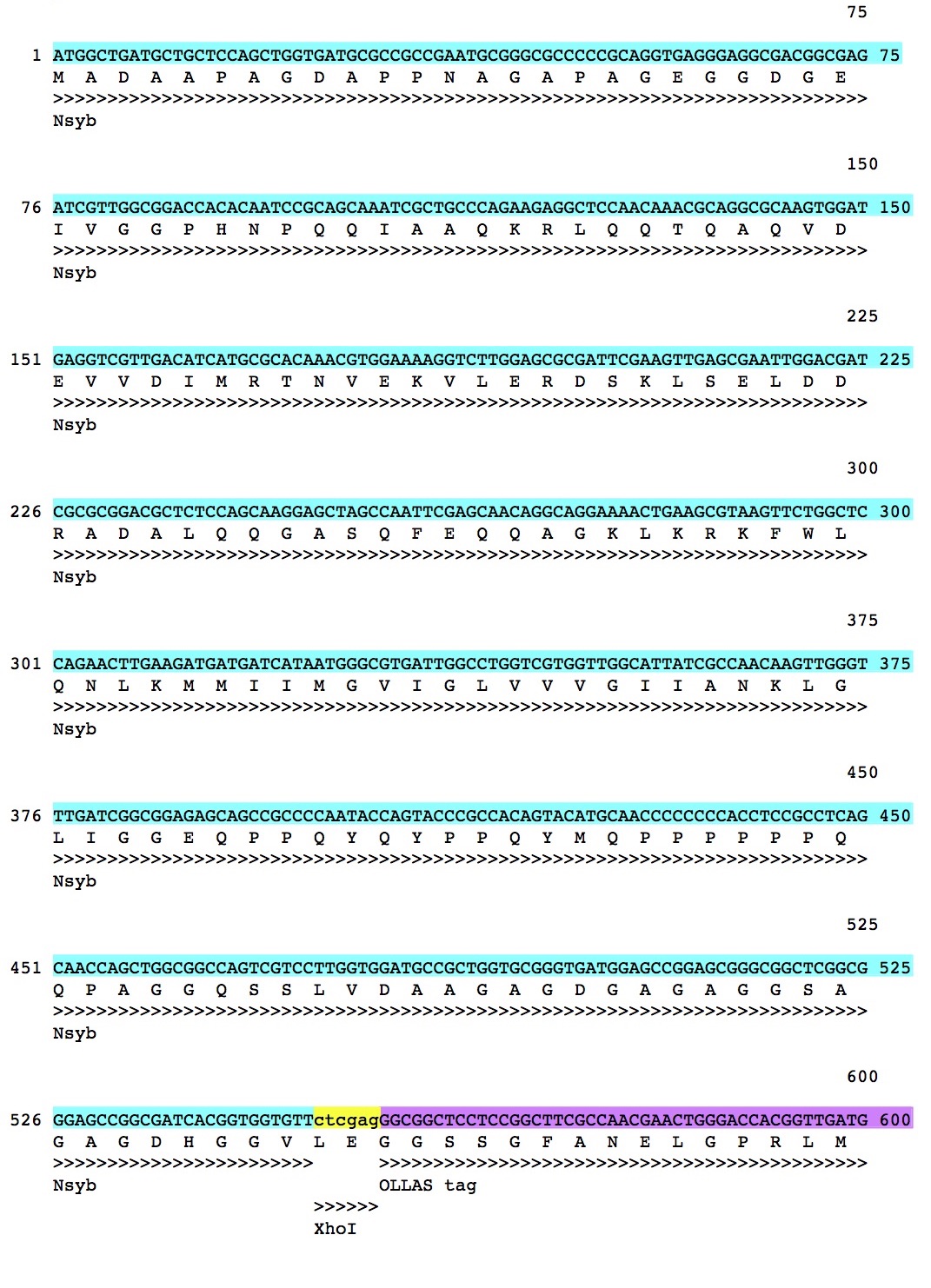


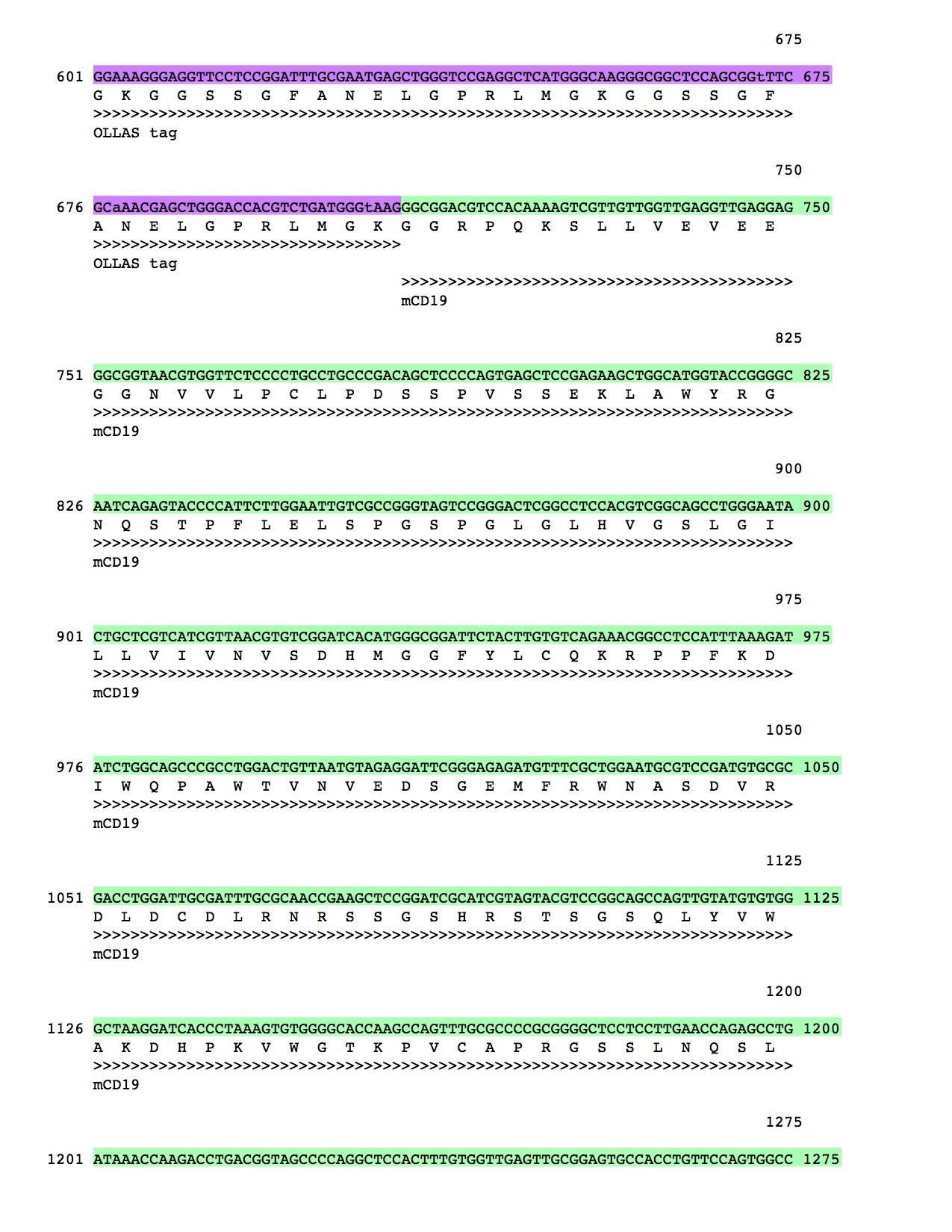

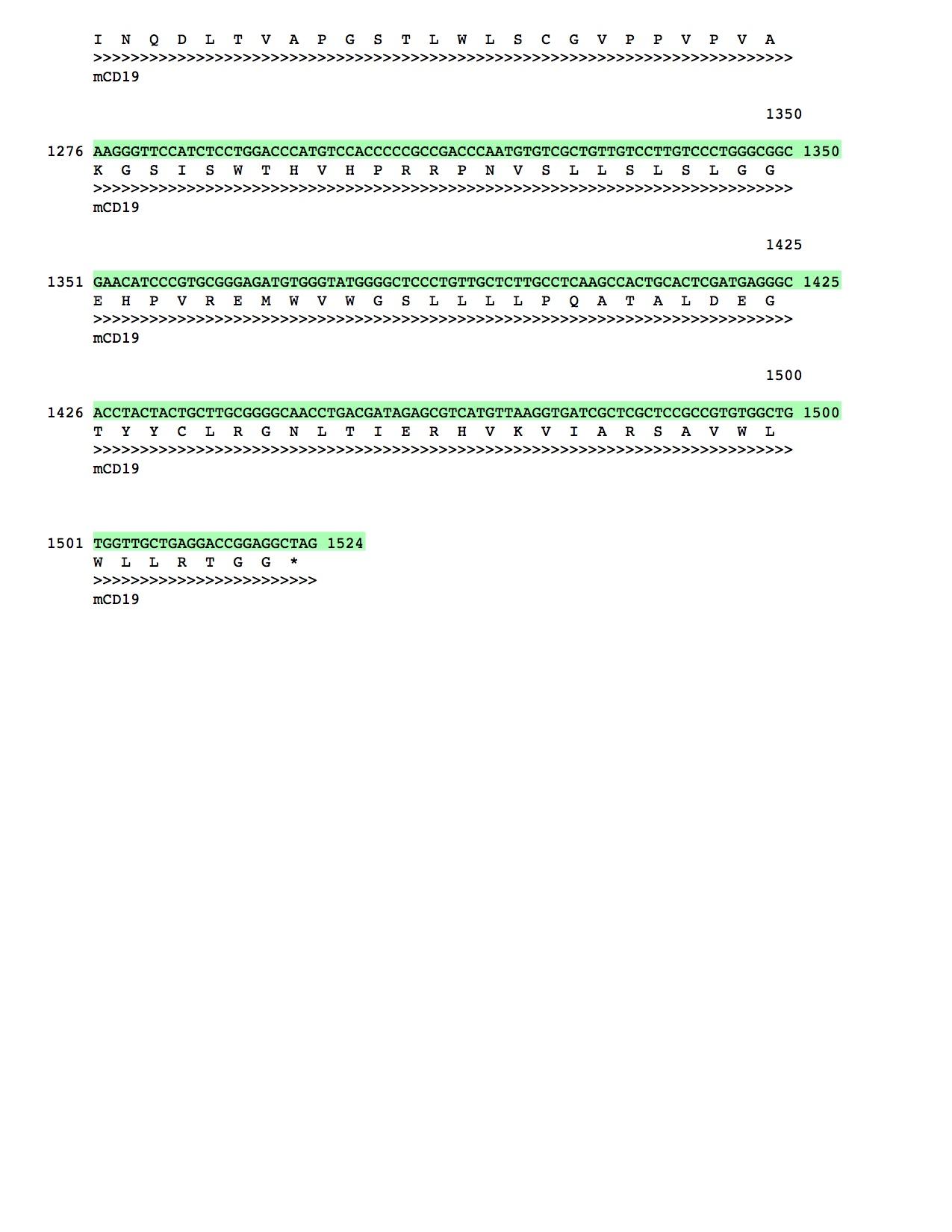


mCD19 sdc 3X OLLAS


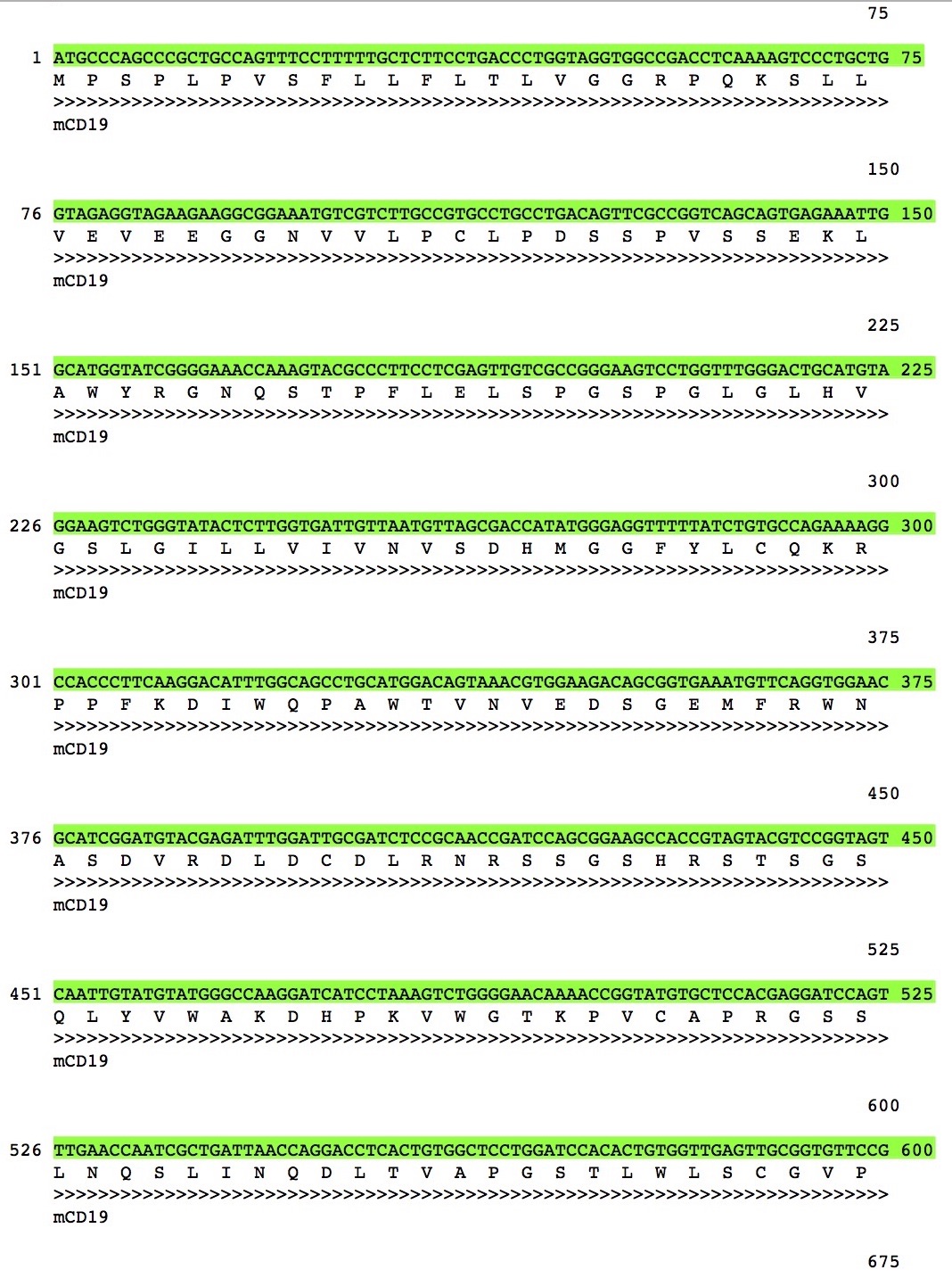


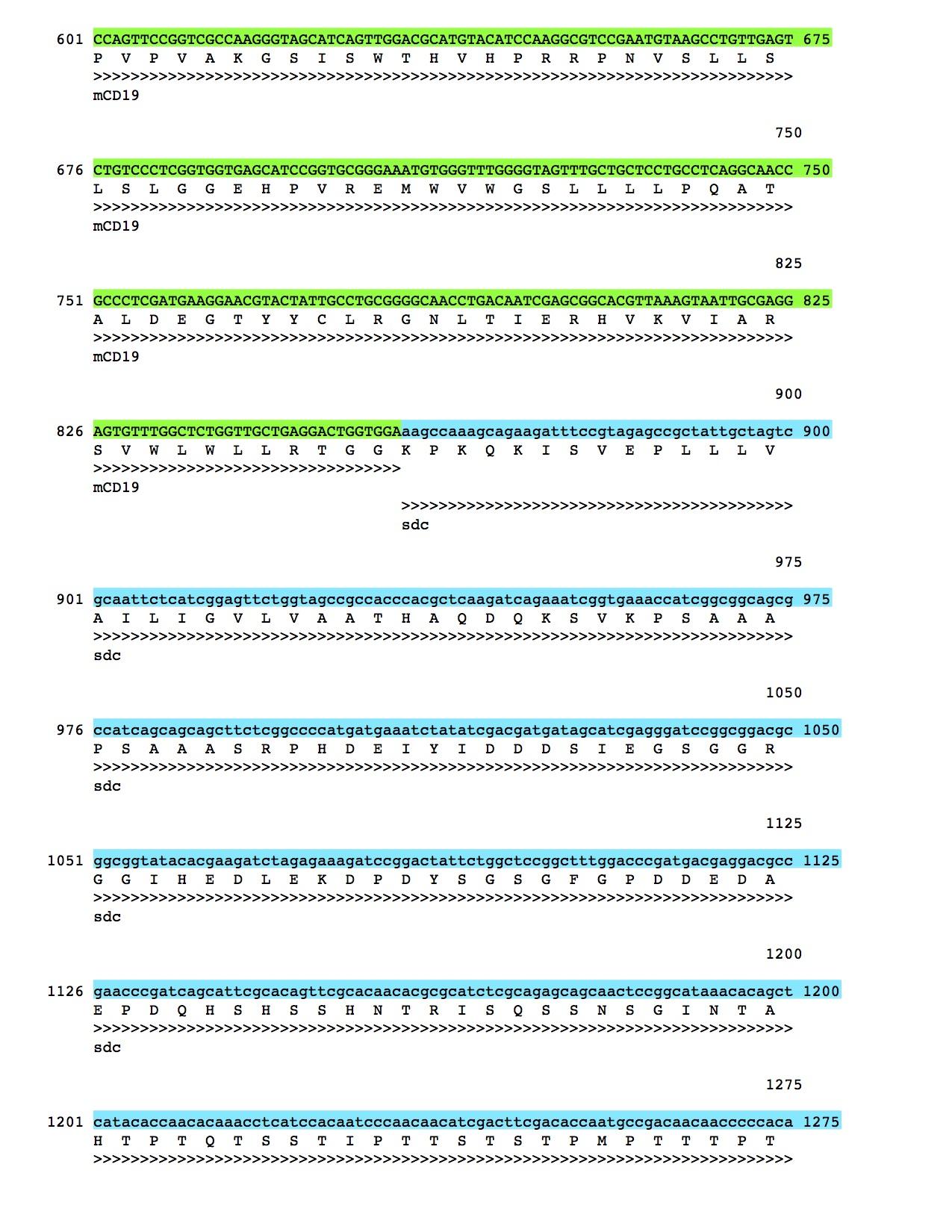

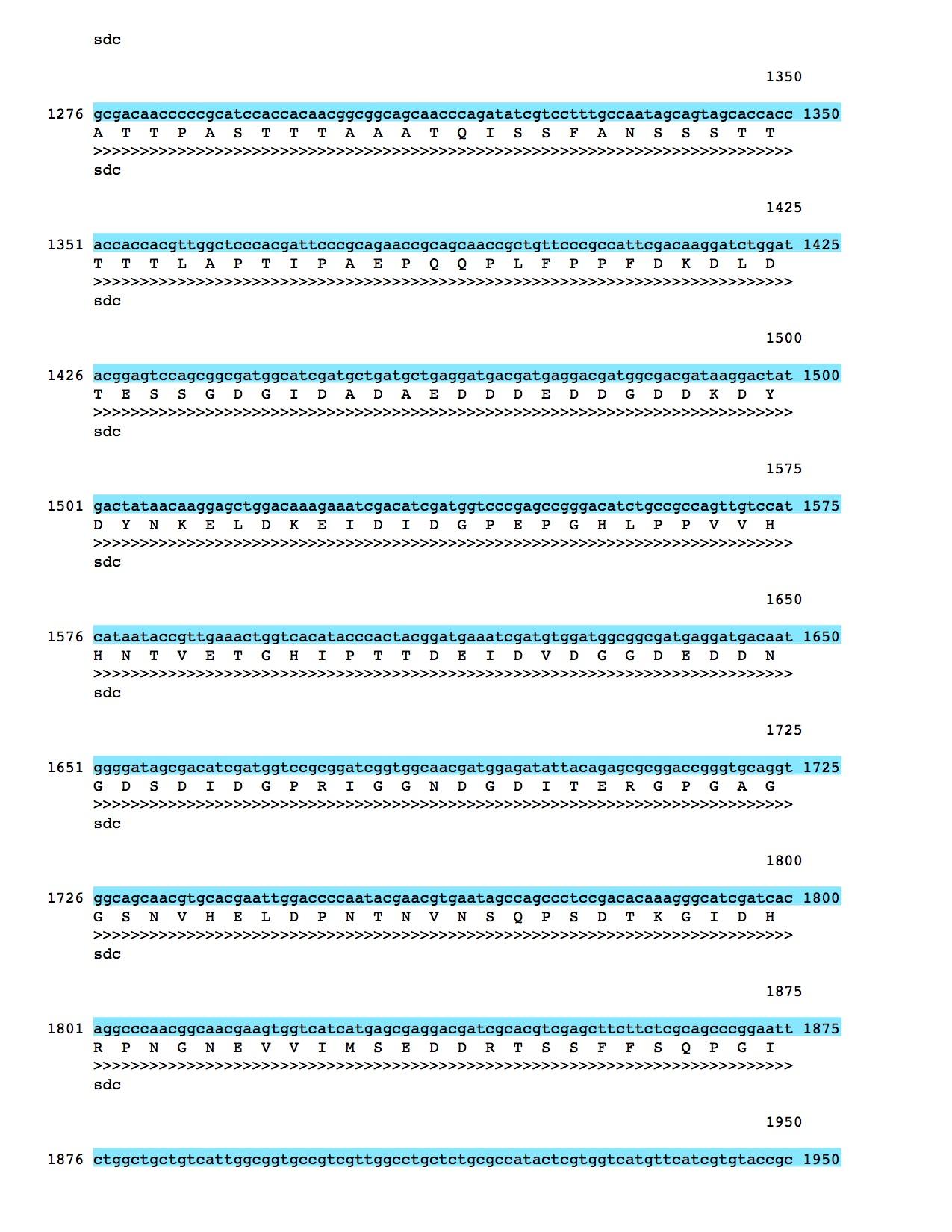

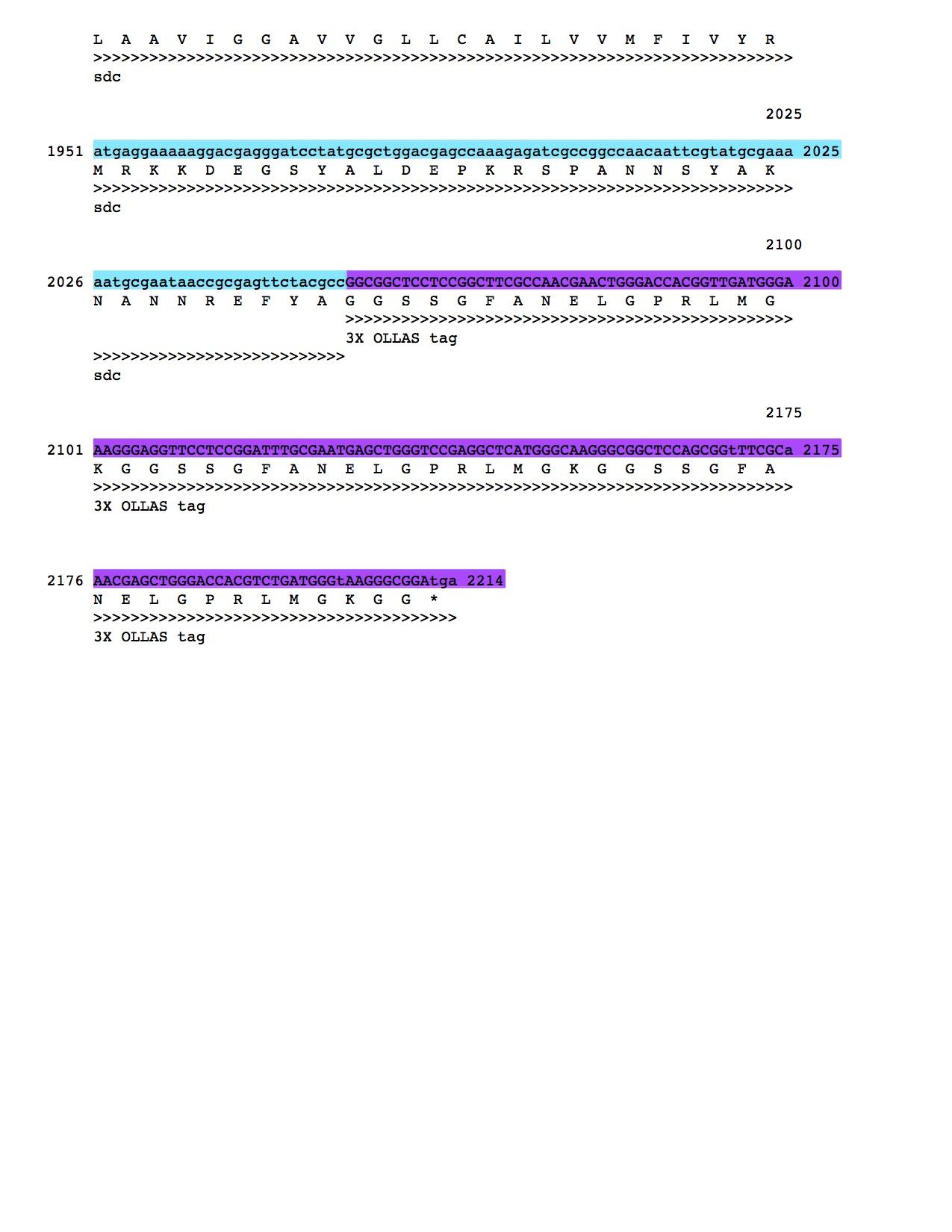

Supplement: Supplementary file 1. [file elife-32027-supp1.docx]
