# Supplementary material for: Tracing neuronal circuits in transgenic animals by transneuronal control of transcription (TRACT)
Source: eLife. 2017 Dec 12;6:e32027. doi: 10.7554/eLife.32027 (PMC5777821; doi:10.7554/eLife.32027)
Supplement: Supplementary file 2. [file elife-32027-supp2.docx]

**Supplementary file 2. Supplementary text for Figure 2.**

Expression of the original TRACT ligand and receptor (without any domains that would localize them to synaptic sites) in neurons failed to reveal any synaptic connections in the antennal lobe.  We hypothesized that, since the ligand and receptor were homogeneously distributed throughout the neuron membrane, only a small fraction of ligand and receptor contained within the small surface area of the synaptic cleft would be in close apposition and available to interact with each other, and the local concentration of each might be insufficient to trigger robust binding. To increase the concentration of the SNTG4 receptor in the postsynaptic sites of receiver neurons, we incorporated the ICD from the neuroligin gene, which in mouse and *C. elegans* has been shown to enrich the localization of molecules into postsynaptic sites (Feinberg *et al.*, 2008; *Kim et al*., 2011).  To this end, we first added the ICD from the *Drosophila* neuroligin (*nlg2*) gene between the TMD and the *esn* of the SNTG4 receptor (Sun *et al*., 2011). Second, we added the V5 epitope after *esn* to facilitate analysis of its subcellular localization (Southern *et al*., 1991). We called this modified receptor nlgSNTG4. Immunostaining against the V5 epitope revealed that, whereas the SNTG4 receptor uniformly distributes throughout the neuronal membrane (data not shown), the nlgSNTG4 is enriched in the neuropil regions, which contain neuronal synapses . To clearly visualize the cell bodies of the activated neurons, we also incorporated a UAS-CD8:GFP reporter which robustly labels the morphology of the entire neuron (Han *et al*., 2011). In the flies carrying *orco>*CD19mch, *elav*>nlgSNTG4, and UAS-CD8:GFP, we observed GFP induction in ~20 neurons in the antennal lobe . We performed immunostaining to identify the cell types that were induced in the antennal lobe and observed that most of the GFP+ neurons (putative postsynaptic targets to the ORNs) were GABA positive (Okada *et al*., 2009), and none of them had axons projecting towards the mushroom body and/or lateral horn. This observation indicates that this implementation of TRACT revealed contacts between ORNs and LNs, but not between ORN to PNs. We obtained the same result with 2 additional *elav*>nlgSNTG4 insertions, indicating that this pattern of transneuronal induction was not due to positional effects (Henikoff, 1990). Two scenarios could account for these observations. First, the *elav* enhancer may not be homogeneously expressed across all neurons; for instance, it may drive stronger expression of the receptor in LNs compared to PNs. Second, the cell-cell interactions between ORNs and LNs may be stronger than those between ORNs and PNs, for example, if ORN-LN unitary connections (the connection between one ORN and one LN) have more and/or larger individual synapses. To overcome the potential limitations of the *elav* driver, we generated new transgenic flies in which the nlgSNTG4 was driven by the *Drosophila* synaptobrevin enhancer (nSybE), a driver that has been successfully used for panneuronal expression in *Drosophila* (Pfeiffer *et al*., 2008; Riabinina *et al*., 2015). To investigate the connectivity originating from ORNs, we generated flies carrying *orco>*CD19mch, nSybE-nlgSNTG4, and UAS-CD4tdGFP, a reporter that allows visualization of the dendrites and axons of neurons (Han *et al*., 2011). In these flies, we observed GFP expression in dozens of LNs and PNs (identifiable by their axons projecting into the mushroom body and lateral horn), consistent with the known connectivity between ORNs and LNs, and ORNs and PNs. To test the ability of TRACT to reveal neurons exclusively connected by synapses, we crossed GH146-nlgSNTG4 flies with flies that expressed the CD19mch ligand in identified glomeruli. If TRACT exclusively revealed neurons connected by synaptic contacts, the uniPNs whose receptors were activated by interaction with the ligand would have GFP+ dendrites that would selectively branch in the glomeruli onto where the ORNs would converge their axons. We crossed the GH146-nlgSNTG4 flies with flies expressing the CD19mch ligand in glomeruli VC1 and DA1/VA6/VA1lm under the enhancers of GMR28H10 and GMR17H02 LexA drivers, respectively ([http://www.virtualflybrain.org](http://www.virtualflybrain.org/)). We observed that whereas the CD19mch+ axons were clearly localized into the expected individual glomeruli, there were CD4::tdGFP+ uniPNs whose dendrites projected to other glomeruli where the CD19mch ligand was not expressed. This observation indicates that in this implementation, the TRACT system reveals neurons that are connected by non-synaptic contacts, and that whereas the domain from the *nlg2* gene enriched the localization of the receptor into the neuropil and increased the sensitivity of TRACT, it was not sufficient to direct the receptor exclusively into synaptic sites. To obtain a system that would exclusively reveal neurons connected by synapses, we attempted to target the ligand into presynaptic sites. To this end, we tested a number of domains from *Drosophila* molecules known to be localized into presynaptic terminals: neurexin (*nrxn*) (Li *et al*., 2007), syndecan (*sdc*) (Spring *et al*., 1994), dip ɣ (*dip*), dpr10 (*dpr*) (Carrillo *et al*., 2015; Tan *et al*., 2015), syntaxin (*syx*) (Schulze *et al*., 1995), and synaptobrevin (*nSyb*) (Sudhof *et al*., 1989). We generated constructs under the LexOP promoter in which different domains from these presynaptic molecules were added onto the CD19 ligand, and included the OLLAS epitope tag to facilitate immunodetection. We generated flies with these constructs, performed immunostaining with an antibody that recognizes the OLLAS tag (Park *et al*., 2008), and observed that all the tested domains were enriched in axons, but only nSyb was exclusively localized into presynaptic sites. Next, we crossed these flies, with presynaptically localized ligands expressed in individual glomeruli (glomeruli VC1 and DA1/VA6/VA1lm), with flies expressing the nlgSNTG4 receptor selectively in PNs (GH146-nlgSNTG4) (Figure 5). We observed that expression of the syx::CD19, CD19::dip, CD19::dpr and CD19::nrxn in identified glomeruli showed induction with fewer PNs than those observed with the non-synaptically localized CD19mch ligand, but still there were uniPNs whose dendrites branched outside of the glomeruli where the ligand was expressed (data not shown). This observation indicates that the *syx*, *dpi*, *dpr*, and *nrxn* domains enriched the localization of the ligand onto axons, but were insufficient to reveal exclusive synaptic contacts between neurons.

Carrillo RA, O¨ zkan E, Menon KP, Nagarkar-Jaiswal S, Lee PT, Jeon M, Birnbaum ME, Bellen HJ, Garcia KC, Zinn K. 2015. Control of Synaptic Connectivity by a Network of Drosophila IgSF Cell Surface Proteins. Cell 163: 1770–1782. DOI: https://doi.org/10.1016/j.cell.2015.11.022, PMID: 26687361

Feinberg EH, Vanhoven MK, Bendesky A, Wang G, Fetter RD, Shen K, Bargmann CI. 2008. GFP Reconstitution Across Synaptic Partners (GRASP) defines cell contacts and synapses in living nervous systems. Neuron 57:353– 363. DOI: https://doi.org/10.1016/j.neuron.2007.11.030, PMID: 18255029

Han C, Jan LY, Jan YN. 2011. Enhancer-driven membrane markers for analysis of nonautonomous mechanisms reveal neuron-glia interactions in Drosophila. PNAS 108:9673–9678. DOI: https://doi.org/10.1073/pnas. 1106386108, PMID: 21606367

Henikoff S. 1990. Position-effect variegation after 60 years. Trends in Genetics 6:422–426. DOI: https://doi.org/ 10.1016/0168-9525(90)90304-O, PMID: 2087785

Kim J, Zhao T, Petralia RS, Yu Y, Peng H, Myers E, Magee JC. 2011. mGRASP enables mapping mammalian synaptic connectivity with light microscopy. Nature Methods 9:96–102. DOI: https://doi.org/10.1038/nmeth. 1784, PMID: 22138823

Li J, Ashley J, Budnik V, Bhat MA. 2007. Crucial role of Drosophila neurexin in proper active zone apposition to postsynaptic densities, synaptic growth, and synaptic transmission. Neuron 55:741–755. DOI: https://doi.org/ 10.1016/j.neuron.2007.08.002, PMID: 17785181

Okada R, Awasaki T, Ito K. 2009. Gamma-aminobutyric acid (GABA)-mediated neural connections in the Drosophila antennal lobe. The Journal of Comparative Neurology 514:74–91. DOI: https://doi.org/10.1002/ cne.21971, PMID: 19260068

Park SH, Cheong C, Idoyaga J, Kim JY, Choi JH, Do Y, Lee H, Jo JH, Oh YS, Im W, Steinman RM, Park CG. 2008. Generation and application of new rat monoclonal antibodies against synthetic FLAG and OLLAS tags for improved immunodetection. Journal of Immunological Methods 331:27–38. DOI: https://doi.org/10.1016/j.jim. 2007.10.012, PMID: 18054954

Pfeiffer BD, Jenett A, Hammonds AS, Ngo TT, Misra S, Murphy C, Scully A, Carlson JW, Wan KH, Laverty TR, Mungall C, Svirskas R, Kadonaga JT, Doe CQ, Eisen MB, Celniker SE, Rubin GM. 2008. Tools for neuroanatomy and neurogenetics in Drosophila. PNAS 105:9715–9720. DOI: https://doi.org/10.1073/pnas.0803697105, PMID: 18621688

Riabinina O, Luginbuhl D, Marr E, Liu S, Wu MN, Luo L, Potter CJ. 2015. Improved and expanded Q-system reagents for genetic manipulations. Nature Methods 12:219–222. DOI: https://doi.org/10.1038/nmeth.3250, PMID: 25581800

Schulze KL, Broadie K, Perin MS, Bellen HJ. 1995. Genetic and electrophysiological studies of Drosophila syntaxin-1A demonstrate its role in nonneuronal secretion and neurotransmission. Cell 80:311–320. DOI: 10.1016/0092-8674(95)90414-X, PMID: 7834751

Southern JA, Young DF, Heaney F, Baumga¨rtner WK, Randall RE. 1991. Identification of an epitope on the P and V proteins of simian virus 5 that distinguishes between two isolates with different biological characteristics. Journal of General Virology 72 (Pt 7):1551–1557. DOI: https://doi.org/10.1099/0022-1317-72-7-1551, PMID: 1713260

Spring J, Paine-Saunders SE, Hynes RO, Bernfield M. 1994. Drosophila syndecan: conservation of a cell-surface heparan sulfate proteoglycan. PNAS 91:3334–3338. DOI: https://doi.org/10.1073/pnas.91.8.3334, PMID: 815 9748

Sudhof TC, Baumert M, Perin MS, Jahn R. 1989. A synaptic vesicle membrane protein is conserved from mammals to Drosophila. Neuron 2:1475–1481. DOI: https://doi.org/10.1016/0896-6273(89)90193-1, PMID: 2560644

Sun M, Xing G, Yuan L, Gan G, Knight D, With SI, He C, Han J, Zeng X, Fang M, Boulianne GL, Xie W. 2011. Neuroligin 2 is required for synapse development and function at the Drosophila neuromuscular junction. Journal of Neuroscience 31:687–699. DOI: https://doi.org/10.1523/JNEUROSCI.3854-10.2011, PMID: 2122817

Tan L, Zhang KX, Pecot MY, Nagarkar-Jaiswal S, Lee PT, Takemura SY, McEwen JM, Nern A, Xu S, Tadros W, Chen Z, Zinn K, Bellen HJ, Morey M, Zipursky SL. 2015. Ig superfamily ligand and receptor pairs expressed in synaptic partners in Drosophila. Cell 163:1756–1769. DOI: https://doi.org/10.1016/j.cell.2015.11.021, PMID: 26687360
